# Ofatumumab for the management of refractory pemphigus vulgaris: A case series

**DOI:** 10.1016/j.jdin.2025.05.018

**Published:** 2025-07-15

**Authors:** Sera Sarsam, Ailin He, Dédée F. Murrell

**Affiliations:** a Department of Dermatology, St George Hospital, Sydney, Australia; b Faculty of Medicine, University of New South Wales, Sydney, Australia

**Keywords:** autoimmune, blistering disease, BTK inhibitors, ofatumumab, pemphigus vulgaris

*To the Editor:* Pemphigus vulgaris (PV) is an autoimmune blistering disease for which rituximab is a first-line Food and Drug Administration-approved treatment.^1^ Rituximab use is limited by the need for infusion suites, long-term irreversible B-cell suppression, and adverse events that may preclude retreatment.^2^ An alternative, effective B-cell suppressing agent is therefore needed. Ofatumumab, a monoclonal antibody targeting a distinct CD20 epitope, exhibits enhanced Fc effector functions compared to rituximab and is Food and Drug Administration-approved for refractory multiple sclerosis and chronic lymphocytic leukemia.^2^^,^^3^ We present the long-term efficacy of ofatumumab in weaning 3 patients with refractory PV off glucocorticosteroids.

## Patient 1

A 42-year-old male presented with buccal mucosa and neck PV refractory to multiple treatments (Table I). He received monthly subcutaneous ofatumumab 20 mg as part of a clinical trial. His initial Pemphigus Disease and Area Index (PDAI) score, while on 20 mg prednisone, was 0. He successfully weaned off prednisone within 16 weeks without relapsing, with Dsg3 antibodies reduced over the 7-month period (Supplementary Figs 1 and 2, available via Mendeley at https://data.mendeley.com/datasets/rdzywjxbbv/1). He remained in complete remission until trial discontinuation, then commenced monthly intravenous ofatumumab 3 months later, maintaining remission. Treatment was discontinued after an infusion reaction, but he remained in clinical remission without immunosuppressants. At the 2023 follow-up, his PDAI score remained 0 with undetectable Dsg3 antibodies.

**Table I:** Summary of patient characteristics, treatment received, and treatment response

Patient number	Age/sex/race	Clinical characteristics/previous treatments	Treatment received	Adjuvant therapy	Treatment response	Adverse events
1	42/M/Māori	PV affecting buccal mucosa and neck since 2006 refractory to high-dose prednisone, mycophenolate, intravenous immunoglobulin (IVIG), and rituximab	Screening: Prednisone 20 mg/d for 2 wk plus randomized to Ofatumumab SC 20 mg monthly (RCT July 2015-March 2016) and Ofatumumab IV 20 mg monthly (June 2016-June 2018)	Prednisone 20 mg/d was weaned off by wk 16 of the trial.	**Screening (2015) –** PDAI-TAS 0 **Baseline:** PDAI-TAS 0 Dsg3 antibody titer 133 U/mL **End of trial (7-mo period):** PDAI -TAS 0 Dsg3 antibody titer 102 U/mL **18 mo posttreatment with IV ofatumumab** PDAI-TAS 0 Dsg3 antibody titer 46 U/mL **Six years posttreatment cessation (2024)** PDAI-TAS 0 Dsg3 antibody titer undetectable	Urticaria and throat tightening on last IV formulation
2	54/F/Chinese	PV affecting oral mucosa since 2011 relapsing after courses of prednisone, rituximab, IVIG, mycophenolate, and dapsone.	Screening: prednisone 20 mg/d for 2 wk Randomized to Ofatumumab SC 20 mg monthly (Oct 2015-March 2016) and Ofatumumab IV 20 mg monthly (July 2016-March 2019), then every 8 wk until March 2021.	Prednisone 20 mg/d was weaned off by wk 16 of the trial.	**Screening:** PDAI-TAS 2 **Baseline:** PDAI-TAS 0 Dsg3 antibody titer: 87 U/mL **End of trial (6-mo period):** PDAI-TAS 0 Dsg3 antibody titer: 33 U/mL **Prior starting IV ofatumumab (Relapse):** PDAI-TAS 4 Dsg3 antibody titer 103 U/mL **Six months post IV treatment initiation:** PDAI-TAS 0 Dsg3 antibody titer undetectable	Urticaria and hypertension on IV formulation
3	50/M/Iraqi	Localized PV to scalp and oral mucosa after responding at other sites to prednisone, mycophenolate, rituximab, phase 2 oral rilzabrutinib, and intralesional corticosteroid injections	Ofatumumab SC 20 mg weekly and Ofatumumab 20 mg intralesional fortnightly	Nil	**Baseline:** PDAI-TAS 1 Dsg3 antibody index 1.67 **Eight months posttreatment initiation:** PDAI-TAS 0 Dsg3 antibody undetectable	Nil reported

Both patients 1 and 2 participated in the OPV116910 trial (Clinical Trials.gov ID NCT01920477).

*IV*, Intravenous; *PDAI*, Pemphigus Disease and Area Index; *PV*, pemphigus vulgaris; *RCT*, randomized controlled trial; *SC*, subcutaneous; *TAS*, total activity score.

## Patient 2

A 54-year-old woman with refractory PV received monthly subcutaneous ofatumumab 20 mg in the same trial (Table I). At baseline, she was on prednisone with a PDAI score of 0 and elevated Dsg3 antibodies. By 16 weeks, she had tapered off prednisone, maintaining a PDAI score of 0 and reduced Dsg3 antibodies (Supplementary Figs 3 and 4, available via Mendeley at https://data.mendeley.com/datasets/rdzywjxbbv/1). After trial discontinuation, she relapsed at 4 months. Therefore, she began compassionate intravenous doses monthly as monotherapy, achieving complete remission with normalized Dsg3 antibodies. In 2019, dosing was extended to every 8 weeks. Treatment was discontinued in 2021 due to COVID-19 after 6 years of remission. Despite two mild infusion reactions, she remained in remission without further prednisone, with sustained Dsg3 reduction 18 months poststeroid treatment.

## Patient 3

**Fig 1:**
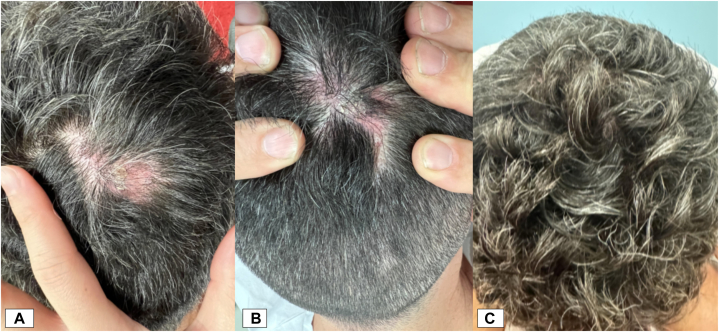
Pemphigus vulgaris. Patient 3 clinical photographs at baseline **(A)**, at 8 weeks **(B)** with significant reduction in size, smaller overlying crust, and evidence of hair regrowth, and at 16 weeks post-ofatumumab initiation **(C)** demonstrating resolution.

A 50-year-old male with refractory oral mucosa and scalp PV, relapsing after multiple therapies, was initiated on compassionate ofatumumab 20 mg (Table I). He received two subcutaneous doses weekly, followed by two intralesional doses fortnightly for a persistent and painful 6 cm biopsy-proven scalp PV lesion. At 8 weeks, the lesion significantly reduced in size with smaller overlying crust and evidence of hair regrowth (Fig 1). Additional intralesional doses were given at 8-week intervals to week 16. Over 8 months, he experienced lesion resolution, hair regrowth, reduced pain, a PDAI score of 0, and undetectable Dsg3 antibodies (Supplementary Fig 5, available via Mendeley at https://data.mendeley.com/datasets/rdzywjxbbv/1). He remained in complete remission 16 months after the last intralesional injection, highlighting ofatumumab’s efficacy in managing localized refractory disease.

Ofatumumab offers enhanced cytotoxicity and a shorter half-life than rituximab, reducing long-term immunosuppression risks.^4^ All patients achieved complete remission off therapy, with reduced Dsg3 antibody and no additional immunosuppressants. Subcutaneous ofatumumab improved safety and convenience, while intralesional administration effectively treated localized lesions. Ofatumumab's shorter B-cell suppression duration is particularly advantageous during pandemics, mitigating infection and vaccination challenges associated with rituximab.^1^^,^^5^ These cases support ofatumumab’s potential as a treatment for PV, warranting further investigation through clinical trials.

## Conflicts of interest

Dr Murrell is an investigator and advisory board member for Principia Biopharma and was a primary investigator and/or advisor for Roche, GSK/Stiefel/Novartis, Principia/Sanofi, JNJ, and Lilly. Drs Sarsam and He have no conflicts of interest to declare.
